# Characterization of temperature‐dependent hemin uptake receptors HupA and HvtA in *Vibrio vulnificus*

**DOI:** 10.1002/mbo3.905

**Published:** 2019-07-10

**Authors:** Shreya Datta, Ryan J. Kenton

**Affiliations:** ^1^ Acumed LLC USA Headquarters Hillsboro OR USA; ^2^ Department of Biology University of Portland Portland OR USA

**Keywords:** Hemin, HupA, HvtA, Iron, *Vibrio vulnificus*

## Abstract

The Gram‐negative pathogen *Vibrio vulnificus* produces several iron‐sequestration systems including a hemin uptake system in response to iron limitation as a means to acquire this essential element. Strains of this organism are capable of causing serious septicemia in humans and eels, where hemin is abundant and an advantageous source of iron. *Vibrio vulnificus* hemin uptake systems consist of HupA, a well studied outer membrane protein, and a recently identified HvtA protein receptor. In this study, we confirmed that the expression of the *hvtA* gene is iron‐regulated in a fur‐dependent manner. When analyzed for virulence in a hemin‐overloaded murine model system, the *hupA* gene was more important for establishing infection than the *hvtA* gene. Transcriptional profiling of these genes using strains of two different biotypes, biotype 1 (human pathogen) and biotype 2 (eel pathogen), showed that the expression of the two receptors was also regulated in response to temperature. The expression of *hupA* was highly induced in elevated temperatures in the human pathogenic strain when tested in iron‐depleted conditions. Conversely, *hvtA* expression was induced significantly in the eel pathogenic strain at a lower temperature, a condition where the *hupA* locus was relatively repressed. Our results indicate that although both *hupA* and *hvtA* are involved for optimal hemin uptake in *V. vulnificus*, their expression is dually regulated by the environmental cues of iron concentration and temperature. Together, these data suggest that the virulence genes *hupA* and *hvtA* are tightly regulated and strictly induced during iron limitation combined with the physiological temperature of the host organism.

## Graphical abstract

This study expands our understanding of the contribution of the *hvtA* locus in *Vibrio vulnificus* to virulence by uncovering an important correlation between gene expression and environmental temperature. This temperature‐mediated regulation is coupled with responsiveness to iron depletion.

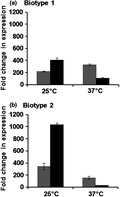

## 1 INTRODUCTION

Many Gram‐negative bacterial pathogens require iron as an essential element for survival. Iron is known to play significant roles in signaling pathways and is used as a cofactor or prosthetic group for several proteins that are required for proper maintenance of cellular functions (Schaible & Kaufmann, 2004; Wandersman & Delepelaire, 2004). Although iron is abundant within the host environment, it exists predominantly in complex with high‐affinity iron‐binding proteins such as lactoferrin and transferrin, or is bound to hemin (Fe^3+^‐heme) in red blood cells, making it essentially unavailable (Crosa, 1997; Koster et al., 1991; Wandersman & Delepelaire, 2004; Wilks & Burkhard, 2007). Therefore, pathogens have evolved to express several iron‐acquisition systems that utilize low molecular weight high‐affinity iron‐chelating siderophores and outer membrane receptors to sequester and acquire the metal (Crosa, 1997; Koster et al., 1991).

*Vibrio vulnificus* is an opportunistic human and marine Gram‐negative bacterial pathogen that may cause fever, diarrhea, and necrotizing wound infections with high mortality rates in humans and eels. It is responsible for almost 95% of all seafood‐related deaths around the world (Haq & Dayal, 2005; Morris, 1988; Strom & Paranjpye, 2000). Additionally, *V. vulnificus* infections can lead to fulminant septicemia in immunocompromised patients where the pathogen invades the bloodstream and causes septic shock (Gulig, Bourdage, & Starks, 2005; Koenig, Mueller, & Rose, 1991; Merkel, Alexander, Zufall, Oliver, & Huet‐Hudson, 2001). The utilization of iron by this bacterium has thus been extensively studied to gain insight into its contribution to pathogenesis. It has been shown to express several iron‐sequestering systems similar to other Gram‐negative species when cultured under iron‐limiting conditions (Litwin & Byrne, 1998; Simpson & Oliver, 1983).

In *V. vulnificus*, iron is acquired through the utilization of two high‐affinity small molecular weight siderophores, the catecholic vulnibactin, and a hydroxamate‐type molecule, both biosynthesized and secreted in response to iron deprivation that serves as iron scavengers from the extracellular surroundings (Litwin, Rayback, & Skinner, 1996; Okujo et al., 1994; Simpson & Oliver, 1983). Certain outer membrane receptor proteins are also co‐expressed that specifically bind to the ferric‐siderophore complexes and mediate internalization to the periplasmic space where the complex is finally transported across the inner membrane of the bacteria. Alternatively, the bacterium also expresses hemin uptake systems that utilize outer membrane protein receptors responsible for internalizing iron‐bound hemin complex scavenged from the host (Datta & Crosa, 2012; Litwin & Byrne, 1998; Litwin & Quackenbush, 2001; Oh, Lee, Lee, & Choi, 2009). The transport and internalization of both the ferric‐siderophore and hemin complexes are energy‐dependent processes mediated by a complex of proteins known as the TonB energy‐transduction system which is located in the inner membrane of the bacteria (Braun, 1995; Crosa, Mey, & Payne, 2004; Postle & Larsen, 2007; Wright, Simpson, & Oliver, 1981). *Vibrio* species and *Vibrionaceae* family members are known to possess two TonB systems (TonB1 and TonB2). Interestingly, *V. vulnificus* has been shown to contain an additional system, TonB3. (Alice, Naka, & Crosa, 2008; Kuehl & Crosa, 2010; Kustusch, Kuehl, & Crosa, 2011; Stork, Otto, & Crosa, 2007) It has also been demonstrated that the TonB2 (and TonB3) systems consist of a fourth protein TtpC apart from the classic ExbB2, ExbD2, and TonB2 (or ExbB3, ExbD3, and TonB3) proteins (Kuehl & Crosa, 2010; Stork et al., 2007). The expression of many of these iron‐regulated virulence factors in *V. vulnificus* is dependent on the regulatory *fur* gene, whose product represses transcription of specific genes when an adequate concentration of iron is present (Litwin & Calderwood, 1993; Miyamoto et al., 2009). Low concentration of iron in host tissues serves as an important signal to direct the expression of virulence factors through the release of the Fur protein from consensus DNA sequences, termed Fur boxes, located in the promoter region of these iron‐regulated genes (Escolar, Perez‐Martin, & Lorenzo, 1999).

*Vibrio vulnificus* strains are divided into three biotypes—1, 2, and 3. Biotype 1 and biotype 3 are known as opportunistic pathogens in humans (Bisharat et al., 1999; Efimov et al., 2013; Hor, Gao, & Wan, 1995). Biotype 2 is primarily an eel pathogen, and only particular isolates have been implicated in human infection (Amaro & Biosca, 1996; Tison, Nishibuchi, Greenwood, & Seidler, 1982). Although biochemically and serologically different, the three biotypes share common virulence features such as flagella, cytotoxin, capsular polysaccharides, and iron‐regulated virulence determinants (Amaro, Biosca, Fouz, & Garay, 1992; Amaro, Biosca, Fouz, Toranzo, & Garay, 1994; Biosca et al., 1993; Kreger, Kothary, & Gray, 1988; Ran Kim & Haeng Rhee, 2003; Simpson, White, Zane, & Oliver, 1987). It has been demonstrated that the capsular material is a necessity for pathogenesis in humans but dispensable for eel infection (Amaro et al., 1994; Biosca et al., 1993; Simpson et al., 1987). Both the biotype 1 and biotype 2 strains have also been shown to utilize hemin and hemoglobin to alleviate iron limitation, indicating that hemin acquisition is an important mechanism for scavenging iron to promote survival of the bacterium (Fouz et al., 1996; Helms, Oliver, & Travis, 1984).

HupA, an outer membrane protein (79.27 kDa), was reported to be a hemin receptor and the expression of the *hupA* gene that encodes the receptor protein was demonstrated to be transcriptionally regulated (a) in response temperature, (b) by the iron‐binding regulatory protein Fur, and (c) a LysR homolog HupR (Litwin & Byrne, 1998; Litwin & Quackenbush, 2001; Oh et al., 2009). We reported the identification of an additional iron‐regulated TonB‐dependent outer membrane hemin receptor HvtA (79.09 kDa), which was shown to facilitate optimum hemin utilization in *V. vulnificus* using the biotype 1 CMCP6 strain (Datta & Crosa, 2012). In this report, we further study and compare the regulation of the *hupA* and *hvtA* genes in biotypes 1 (CMCP6) and 2 (ATCC33149) *V. vulnificus* strains and demonstrate that they are dually regulated in response to iron concentration and temperature at the transcriptional level. Further, we hypothesize that apart from the *hupA* gene, the *hvtA* gene may also play an essential role in the bacterium's pathogenicity with individual requirements specific to the environment of the host organism (37°C in humans and 25°C in eels) where each gene is upregulated.

## 2 MATERIALS AND METHODS

### 2.1 Bacterial strains, plasmids, and growth conditions

Strains and plasmids used in this study are listed in Table 1. Bacteria were routinely grown in trypticase soy broth supplemented with 1% NaCl (TSBS) or on trypticase soy agar supplemented with 1% NaCl (TSAS; *V. vulnificus*), or in LB broth (*E. coli*) with appropriate antibiotics: kanamycin (50 μg/ml) and chloramphenicol (30 μg/ml) for *E. coli* unless otherwise mentioned. M9 minimal medium (Crosa, 1980) was used for iron‐limiting conditions supplemented with 0.2% casamino acids and 5% NaCl with the iron chelator ethylenediamine‐di‐(o‐ hydroxyphenylacetic) acid (EDDA) at indicated concentrations. Ferric ammonium citrate (FAC) was added to the medium to obtain iron‐rich growth conditions at indicated concentrations. Thiosulfate‐citrate‐bile‐salts‐sucrose agar (TCBS; Preiser Scientific, Louisville, KY) was used for selection of *V. vulnificus* in conjugation experiments.

**Table 1:** Strains and plasmids used in this study

Strain or plasmid	Genotype or relevant characteristic	Source or reference
*V. vulnificus*		
CMCP6	Wild‐type clinical isolate of *V. vulnificus*	J. Rhee
AA‐16	Δ*venB*, Δ*tonB1*, Δ*tonB2*, Δ*tonB3*	Alice et al. (2008)
AA‐14	Δ*venB*	Alice et al. (2008)
VSSD101	Δ*venB*Δ*hvtA*Δ*tonB1*	This study
VSSD103	Δ*venB*Δ*hvtA*Δ*tonB2*	This study
VSSD107	Δ*venB*Δ*hupA*Δ*tonB1*	This study
VSSD113	Δ*venB*Δ*hupA*Δ*tonB2*	This study
ALE‐LAC	Δ*lacZ*	Alice et al. (2008)
VSSD58	Δ*hvtA*	This study
VSSD86	Δ*hupA,* Δ*lacZ*	This study
VSSD74	Δ*hupA,* Δ*hvtA*	Datta and Crosa (2012)
AA‐2	Δ*lacZ fur*::pDM4	Alice et al. (2008)
VSSD59	Δ*hupA*	This study
VSSD100	Wild‐type biotype 2 ATCC 33,149 *V. vulnificus* strain	Tison et al. (1982)
*E. coli*		
TOP10	F^‐^ *mcrA Δ(mrr‐hsdRMS‐mcrBC) Φ*80*lacZΔM15 ΔlacX74 recA1 araD139 Δ(*ara‐leu) *7697 galU galK rpsL* (Str^r^) *endA1 nupG*	Invitrogen
S17‐1λpir	*Λpir* lysogen; *thi pro hsdR hsdM* + *recA* RP4−2 Tc:Mu‐Km:Tn7(Tp^r^ Sm^r^)	Simon, Priefer, and Puhler (1983)
Plasmids		
PCR II Blunt	Blunt‐end cloning vector; Km^r^	Invitrogen
pPCR2.1	TA cloning vector; Amp^r^, Km^r^	Invitrogen
pDM4	Suicide vector with oriR6K; Cm^r^ *sacB*	Milton et al. (1996)
pRK2013	Helper plasmid; Km^r^	Figurski and Helinski (1979)

### 2.2 DNA manipulations and sequence analysis

Plasmid DNA was extracted using the Qiagen Miniprep Kit (Qiagen, Valencia, CA). Genomic DNA was isolated from the *V. vulnificus* CMCP6 and ATCC 33149 strains using the DNeasy^®^ Blood & Tissue Kit (Qiagen). Polymerase chain reactions (PCRs) were carried out using a MyCycler™ Thermal Cycler as specified by the manufacturer (Bio‐Rad Laboratories, Hercules, CA). Touchdown PCRs were performed using Vent Polymerase (New England Biolabs Inc., Ipswich, MA), under the following conditions: 95°C for 4 min, 30 cycles of 95°C for 30 s, 63°C for 1 min (temperature of this step was decreased by 0.3°C at each cycle), and 72°C for 1 min, and an extension step of 72°C for 10 min. DNA sequencing reactions were carried out by the Oregon Health and Science University Vollum DNA Sequencing Core Facility. Primers used to amplify the *hupA* (HER1 and HER2) and *hvtA* (VV21549‐1fwd and VV21549‐2rev) genes from the ATCC 33149 biotype 2 *V* *. vulnificus* strain were designed using the analogous gene sequences in the CMCP6 strain (Table 2).

**Table 2:** Primers used in this study

Primer name	Nucleotide sequence (5’−3’)
Amplification of genes in ATCC 33149 strain	
HER1	AATTCGCTTTGTGGCCAGAACGAT
HER2	GTTCATCAATGATACCTTTGGTCA
VV21549‐1fwd	GTTCCAGTCACGCTGGCGTAC
VV21549‐2rev	CTGCAGCGCTTGCAGATCCGC
RT‐PCR	
VV21546_1000fwd	ATGTACACCGTATTGGTCGTAC
VV21548_261rev	CTTGGTTGATTAGCTTCCATTC
VV21548_1700fwd	TGAGCAGCAATATGGAGAATGG
VV21549_285rev	CATCGCGAACGATCATAATTC
VV21549_1771fwd	ATGACCAAGGCGAATACATTCG
VV21550_300rev	CGCCATTGAGCAACACCGTGT
VV21550_1900fwd	CATTTATCTCGATATTGACCAC
VV21551_200rev	GCCATCCAACGCGATGGCGCT
VV21551_1080fwd	CAGCAAACGTTGTGTGGACAA
VV21552_240rev	CTTGCGCCGTTTGCAACGCACT
qRT‐PCR	
B1HupAForqRTPCR	TGATTCAGCATTCACAGGTCG
B1HupARevqRTPCR	GTTAGTGTAACCATGTCCCGG
B1HvtRForqRTPCR	TGATTCGTTTGAGGTAGGGC
B1HvtRRevqRTPCR	CCGTTGAGGTTTTGGTATTGC
B2HupAForqRTPCR	ACTCAGCATTCACAGGTCG
B2HupARevqRTPCR	CCATGTCCCGGATTATCATAGG
B2HvtRForqRTPCR	AAGGATCTGACGTTGAGTGC
B2HvtRRevqRTPCR	CGGCTCTGTGGAATAAGTGG

### 2.3 Construction of *V. vulnificus* mutants

Deletion mutants in *V. vulnificus* CMCP6 strain were generated by allelic exchange using the pDM4 suicide plasmid (Milton, O'Toole, Horstedt, & Wolf‐Watz, 1996). Upstream and downstream regions (700–800 bp) flanking the genes were amplified by specific primers, and combined using splicing by overlapping extension (SOE) PCR. The generated PCR products were cloned into the blunt PCR2.1 vector (Invitrogen, Carlsbad, CA), digested with restriction enzymes, and subcloned into the suicide vector pDM4 also linearized with the same restriction enzymes. The resulting pDM4 derivatives were conjugated into *V. vulnificus* according to the procedure previously reported using the helper plasmid pRK2013 (Alice et al., 2008; Figurski & Helinski, 1979).

### 2.4 Hemin utilization assay

Bioassays were performed to determine whether hemin and hemoglobin could be utilized by the *V. vulnificus* wild‐type and mutant strains. 50 μl from overnight cultures of the bacterial strains was mixed with 20 ml of M9 media containing agar supplemented with 20 μM EDDA and poured into petri dishes. After solidification, different iron sources, FAC: 1 mg/ml (Sigma‐Aldrich, MO), aerobactin: 1 mg/ml (EMC Microcollections, Germany), ferrioxamine: 1 mg/ml (EMC Microcollections, Germany), hemin: 10 μM in 10 mM NaOH (Sigma‐Aldrich), and hemoglobin: 50 μM in 1X phosphate‐buffered saline (PBS; Sigma‐Aldrich) were spotted on the plate and incubated at 37°C, and appearance of the growth halo was monitored after 18 hr.

### 2.5 Virulence assays

Overnight cultures of *V. vulnificus* strains were inoculated into 25 ml of TSBS (inoculation ratio 1:100) and grown at 37°C to an OD_600_ of ~0.5. The cells were then harvested and washed twice in phosphate‐buffered saline (PBS). Next, cells were resuspended to an OD_600_ of 1.0 and serially diluted in PBS. Five 4‐ to 6‐week‐old CD1 mice (Charles River Laboratories) per dilution were injected intraperitoneally (i.p.) with 100 μl of the strain of interest. Five serial dilutions for each strain were evaluated. Mortality was monitored for 48 hr post‐infection, and 50% lethal dose (LD_50_) calculations were determined by the Reed–Muench method (Reed & Muench, 1938). For hemin‐overloaded models, 100 μl of hemin (10 mg/ml) solution prepared in 10 mM NaOH was injected (i.p.) into the animals two hours before injecting the bacterial strains of interest. All manipulations of mice were approved by the Institutional Animal Care & Use Committee (IACUC) at the Oregon Health Science University, protocol #A802.

For Competitive Index (CI) experiments, two strains, VSSD58 (CMCP6:*ΔhvtA*) and VSSD74 (CMCP6: *ΔhupA, ΔhvtA*), were used to compare their CI with ALE‐LAC (CMCP6:*ΔlacZ*) and VSSD86 (CMCP6: *ΔhupA, ΔlacZ*), respectively, to confirm whether the strains behaved similarly in the host. Briefly, bacterial strains were cultured as described above, and then, equal volumes were mixed and serial dilutions were performed. Intraperitoneal injections of three serial dilutions were put into three to six mice. Animals were checked approximately 9 hr onwards after inoculation, and when they showed signs of sickness (e.g., lethargy, slow movements, and lack of appetite), they were euthanized with CO_2_ according to IACUC regulations. Since i.p. injections involve puncture of the skin, skin samples (one square centimeter around the inoculation site) along with spleens, and livers were aseptically extracted and homogenized by using a Seward stomacher laboratory blender in the presence of PBS (1 ml for skin and spleen and 2 ml for liver). Serial dilutions were performed in PBS, and the dilutions were plated on TSAS‐5‐bromo‐4‐chloro‐3‐indolyl‐β‐D‐galactopyranoside (TSAS‐X‐Gal; 0.004%, wt/vol) plates to determine the CFU of the strains under analysis. CI values were obtained as described previously (Taylor, Miller, Furlong, & Mekalanos, 1987). Statistical analysis was performed using the Student *t* test with GraphPad Prism 4.0 software.

### 2.6 RNA extraction and transcript analysis

Overnight cultures of *V. vulnificus* strains were separately inoculated (inoculation ratio 1:200) in M9 minimal media supplemented with 50 μg/ml FAC (for iron‐rich condition) or 2 μM EDDA (for iron‐limiting condition) and grown at 25°C and 37°C to optical densities of 0.4–0.5 at 600 nm. Cells were harvested and total RNA was extracted using RNeasy^®^ Mini Kit (Qiagen) from each sample according to the manufacturer's instructions. Following extraction, total RNA was subjected to DNase treatment with TURBO DNA‐free™ Kit (Ambion, Austin, TX) to remove residual DNA.

For analyzing transcripts, cDNA synthesis was performed using SuperScript II reverse transcriptase (Invitrogen) according to manufacturer's protocols using the provided random primers. Reverse transcriptase PCRs (RT‐PCRs) were then performed using 2 μl of each reverse transcription (RT) reaction mix using gene‐specific primers described in Table 2. A control without reverse transcriptase enzyme in the RT reaction mix was used for each PCR. Fragments were resolved by electrophoresis on agarose gels.

Quantitative RT‐PCRs (qRT‐PCR) were performed using the Power SYBR^®^ Green PCR Master Mix (Applied Biosystems, Foster City, CA) to quantify the expressions of *hupA* and *hvtA* genes with primers listed in Table 2 for biotype 1 (CMCP6) and biotype 2 (ATCC 33149) strains. The levels of mRNA expression of both genes were calculated in iron‐rich and iron‐limiting conditions by normalizing to glyceraldehyde‐3‐phosphate dehydrogenase (GAPDH) expression. Relative fold changes in transcript levels were then calculated between the two growth conditions.

## 3 RESULTS

### 3.1 TonB specificities of the *V. vulnificus* hemin receptors

Prior studies demonstrated that hemin and hemoglobin transport are *tonB1‐* and *tonB2*‐dependent, where *tonB2* also requires *ttpc2* in the *V. vulnificus* CMCP6 strain (Alice et al., 2008; Datta & Crosa, 2012). This indicates that the TonB systems may have redundant functions in transporting iron sources. To determine whether the hemin and hemoglobin receptors HupA and HvtA exhibited specificities for the TonB systems, a series of receptor mutants were generated in the same strain in combination with the individual TonB systems (VV21614 & VV20360). Hemin and hemoglobin uptake assays were then performed using the combinatorial mutant strains described in Table 3. It was previously demonstrated that both the TonB1 and TonB2 systems facilitate ferrioxamine uptake in *V. vulnificus*, whereas the transport of aerobactin was only facilitated by the TonB2 system (Kustusch, Kuehl, & Crosa, 2012). Thus, ferrioxamine and aerobactin were used as controls for the iron utilization assay to ensure the functionality of each TonB system. We have previously shown that only the HupA receptor is capable of transporting hemoglobin (Datta & Crosa, 2012). In addition, it has been shown that the endogenous siderophore, vulnibactin, is capable of stealing iron from both heme and hemoglobin. For these reasons, a *venB* (vulnibactin) mutation is used in combination with our reporter strains. The reporter strains VSSD101 (*ΔvenBΔhvtAΔtonB1*) and VSSD103 (*ΔvenBΔhvtAΔtonB2*) exhibited capacity for hemin and hemoglobin uptake, while the reporter strains VSSD107 (*ΔvenBΔhupAΔtonB1*) and VSSD113 (*ΔvenBΔhupAΔtonB2*) exhibited capacity for only hemin uptake. These results suggest that both HupA and HvtA can utilize either the TonB1 or TonB2 system. In addition, while HupA can mediate both hemin and hemoglobin acquisition, HvtA can only mediate hemin uptake.

**Table 3:** Bioassay to test TonB dependency of the hemin receptors in *V. vulnificus*

Strains	Growth on indicated iron sources^a^				
	FAC	Aerobactin	Ferrioxamine	Hemin	Hemoglobin
Δ*venB*, Δ*tonB1*, Δ*tonB2*, Δ*tonB3*	+	−	−	−	−
Δ*venB*	+	+	+	+	+
Δ*venB*Δ*hvtA*Δ*tonB1*	+	+	+	+	+
Δ*venB*Δ*hvtA*Δ*tonB2*	+	−	+	+	+
Δ*venB*Δ*hupA*Δ*tonB1*	+	+	+	+	−
Δ*venB*Δ*hupA*Δ*tonB2*	+	−	+	+	−

a Two microliters of different iron sources (FAC, 1 mg/ml; aerobactin, 1 mg/ml; ferrioxamine, 1 mg/ml; hemin, 10 μM; and hemoglobin, 50 μM) was spotted on plates containing the various strains, and growth halos were monitored after 18 hr. FAC was used as a positive control because it does not require active transport. +, growth after 18 hr; −, no growth after 18 hr.

### 3.2 Cloning the hemin receptor genes in *V. vulnificus* biotype 2 strain

**Figure 1:**
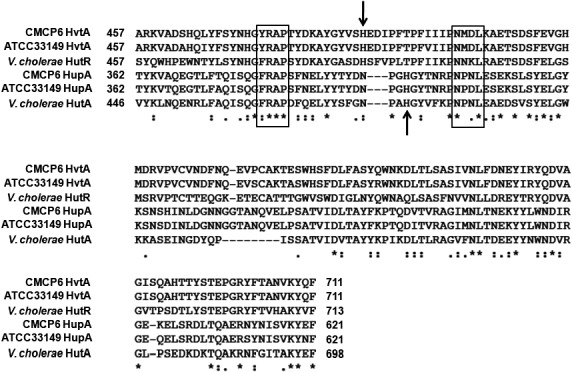
Sequence alignments of HvtA, HupA, and their homologs. A CLUSTALW sequence alignment using HvtA and HupA from CMCP6 (biotype 1), ATCC 33,149 (biotype 2), and the HutA and HutR hemin receptor proteins of *V. cholerae* are shown. Numbering refers to the amino acid position in the primary sequence of the respective proteins. Conserved residues are marked with an asterisk (*), similar residues are annotated by a period (.), and a colon (:) indicates conservation between groups of strongly similar properties. The FRAP and NPNL residues are boxed with the conserved histidine shown with arrows. The terminal phenylalanine (F) is located at the C‐terminus of all proteins

The biotype 2 ATCC33149 strain of *V. vulnificus* has been shown to be extremely infectious in eels (Tison et al., 1982). Since this biotype can also utilize hemin as a source of iron, it was of interest to determine whether homologs of the *hupA* and *hvtA* genes existed in the *V. vulnificus* biotype 2 ATCC33149 strain as the genomic sequence of this strain is not yet published (Gulig et al., 2010; Tison et al., 1982). PCR was performed using primers designed from the genome sequence of CMCP6 biotype 1 strain (Table 2), and the yielded products were subsequently cloned into the pCR2.1 cloning vector and sequenced. Primary sequence analysis of the genes encoding the HupA and HvtA homologs in the ATCC33149 biotype 2 strain indicated open reading frames encoding proteins of 621 (69.43 kDa) and 711 (79.04 kDa) amino acids, respectively. When analyzed, they showed significant identity with the respective genes in the biotype 1 CMCP6 strain both at transcriptional (96% and 95%, respectively) and at translational (97% in both cases) levels. Subsequent sequence analysis in the biotype 1 and biotype 2 strains indicated that HupA and HvtA proteins were significantly homologous in their carboxyl‐terminal ends to the hemin receptor proteins HutA (50% identical, 67% similar) and HutR (51% identical, 68% similar) that are found in *V. cholerae*. Both HupA and HvtA contain a terminal phenylalanine that is indicative of proteins localized in the outer membrane (Figure 1; Struyve, Moons, & Tommassen, 1991). Additionally, they contain conserved FRAP and NPNL box motifs, indicative of membrane receptors specific to hemin. Furthermore, a highly conserved histidine residue between the two motifs was found and has been shown to be significant for binding to and facilitating hemin uptake (Bracken, Baer, Abdur‐Rashid, Helms, & Stojiljkovic, 1999).

### 3.3 Transcriptional analysis of the putative *hvtA* operon

**Figure 2:**
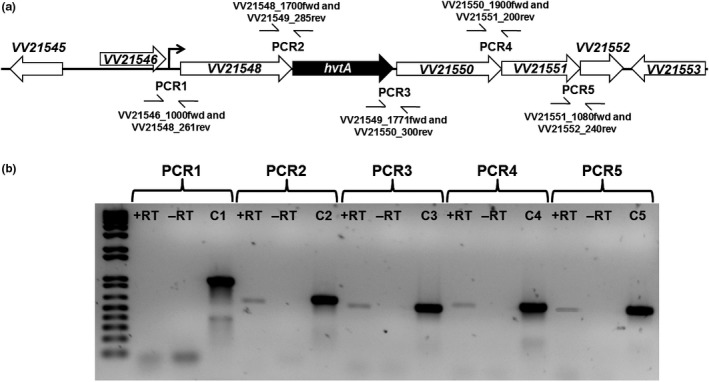
Characterization of the *hvtA* operon in *V. vulnificus*. (a) Schematic map of the *hvtA* operon in *V. vulnificus*. Black and white thick arrows show the ORFs along with their orientations, and the thin right‐angle arrow shows the putative promoter of the operon. The primer locations used in the RT‐PCR analysis (b) are seen between the ORFs in this schematic. (b) RT‐PCR was performed with (+RT) or without (−RT) reverse transcriptase using the primer pairs shown in part A (PCR1‐5). Lanes C1‐C5 show PCR product using CMCP6 genomic DNA as template

DNA sequence analysis of the *hvtA* gene, located in the second chromosome of the CMCP6 strain, suggested that it was positioned as the second open reading frame (ORF) in an operon (Figure 2a). This putative operon is very similar to an operon in *V. cholerae* that contains the hemin receptor gene, hutR (Mey & Payne, 2001). To determine whether the *hvtA* gene was expressed as part of an operon, RT‐PCR was implemented. Briefly, cDNA was generated by growing the wild‐type *V. vulnificus* CMCP6 strain to stationary phase with the provided random oligonucleotides (see Materials and Methods). PCRs 1–5 depicted in Figure 2a,b were performed using the RT mixture with oligonucleotide primers specific to each gene predicted in the *hvtA* operon. The resulting PCR products indicated that the operon contained the ORFs encoding a type II protease gene *ptrB* (VV21548), the *hvtA* gene, and another three ORFs (VV21550‐2), annotated as hypothetical proteins with unknown function. Lack of PCR products obtained from the PCR 1 reaction suggested that gene VV21546 was not a part of the *hvtA* operon. Additionally, these data suggested that the transcriptional start site for the *hvtA* operon was located between the VV21546 and VV21548 genes.

**Figure 3:**
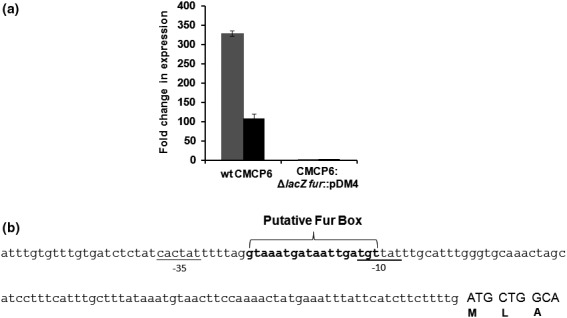
The HvtA operon is transcriptionally regulated by iron. (a) Quantitative real‐time PCR (qRT‐PCR) analysis of the *hupA* (gray bar) and *hvtA* (black bar) genes in biotype 1 wild‐type CMCP6 and CMCP6: Δ*lacZ fur*::pDM4 *V. vulnificus* strains. Strains were grown at 37°C. Fold changes in expression are presented relative to the amount of transcripts yielded in iron‐limiting conditions when compared with iron‐rich conditions. Data are the average of three biological replicates, and error bars represent standard deviation. (b) Nucleotide sequence analysis in the intergenic region between the *VV21546* and *VV21548* genes shows the putative Fur box sequence (bold) and the predicted −10 and −35 regions (underlined). The translational start site of the product of the *VV21548* gene is indicated by the ATG codon including the first three amino acids at the N‐terminal end of the protein

A fur mutant (fur::pDM4) in the CMCP6 strain was grown in minimal M9 media under iron‐rich (10 μg/ml ferric ammonium citrate [FAC]) or iron‐depleted (2 μM EDDA) conditions to log phase at 37°C along with the wild‐type CMCP6 strain (Alice et al., 2008). Levels of *hvtA* mRNA were then determined using quantitative RT‐PCR (qRT‐PCR). The *hupA* mRNA levels were also compared and served as a control for the assay. While both *hupA* expression and *hvtA* expression were elevated when the wild‐type strain was grown under iron‐limiting conditions, no changes in their mRNA levels were detected in the fur mutant strain, suggesting that the *hvtA* gene in the operon was regulated by iron at the transcriptional level (Figure 3a). Similar results were recently reported in the *V. vulnificus* strain M2799 (Kawano et al., 2018). Additionally, detailed analysis of the nucleotide sequence in the *hvtA* operon revealed a potential Fur binding site in the intergenic region between genes VV21546 and VV21548 on the CMCP6 genome, which is the putative transcriptional start site of the *hvtA* operon (Figure 3b). A similar Fur box sequence was recently found in another *Vibrio* strain, *V. vulnificus* M2799, before the *hvtA* operon (Kawano et al., 2018).

### 3.4 Transcription of *hupA* and *hvtA* is impacted by temperature

It was reported that the expression of the *hupA* gene in a human pathogenic *V. vulnificus* strain was influenced by elevated temperature (Oh et al., 2009). The effect of temperature on *hvtA* gene expression has not been studied to date. As *V. vulnificus* is an opportunistic pathogen for both humans and fishes, we sought to determine the expression profiles for both genes in two different strains. Biotype 1 (CMCP6) is a human pathogen, and biotype 2 (ATCC33149) is predominantly an eel pathogen (Amaro & Biosca, 1996; Fouz, Larsen, & Amaro, 2006; Roig & Amaro, 2009). Gene expression profiles were measured and compared after growth at the physiological temperatures of the hosts, 37°C and 25°C, respectively, between iron‐rich (M9 minimal media with 10 μg/ml FAC) and iron‐limiting (M9 minimal media with 5 μM EDDA) conditions. qRT‐PCR analysis revealed the *hupA* and *hvtA* genes were upregulated in iron‐limiting conditions at both temperatures in both biotypes.

**Figure 4:**
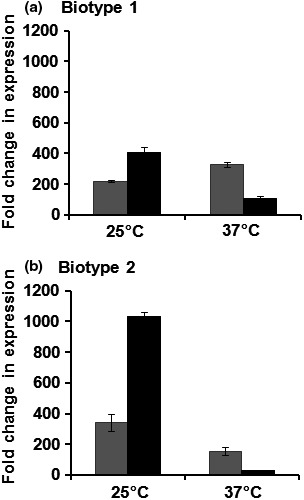
Quantitative RT‐PCR analysis of the *hupA* (gray bar) and *hvtA* (black bar) genes in *V. vulnificus* strains when grown at 25°C and 37°C. Comparison of the *hupA* and *hvtA* gene expression in (a) biotype 1 CMCP6 strain and (b) biotype 2 ATCC 33149 strain. Fold changes in expression are presented relative to the amount of transcripts yielded in iron‐limiting conditions when compared with iron‐rich conditions. Data are the average of three biological replicates and error bars represent standard deviation

It was also interesting to note that the *hupA* gene was more upregulated (~375‐ and 200‐fold) at the higher temperature of 37°C (in both biotype 1 and biotype 2 strains, respectively) than the *hvtA* gene (Figure 4a,b). This observation was consistent at the translational level—while the HupA protein was detected in outer membrane fractions when the biotype 1 CMCP6 strain was cultured at 37°C, HvtA was only detected when overproduced in trans (Datta & Crosa, 2012). Contrastingly, an increase in the expression of *hvtA* mRNA was observed at the lower temperature (25°C) in both biotypes 1 and 2 by ~400‐ and ~1,000‐fold, respectively. Upregulation of the *hvtA* gene was significantly higher at 25°C in the biotype 2 than in the biotype 1 strain (~2.5‐fold), emphasizing its importance at lower temperatures.

### 3.5 The *hupA* and *hvtA* genes have variable contributions in *V. vulnificus* mouse virulence

The requirements for the *hupA* and *hvtA* genes in biotype 1 *V. vulnificus* virulence were assessed by comparing the wild‐type CMCP6 bacterial strain with the hemin receptor mutants in a murine model system using 4‐ to 6‐week‐old CD‐1 normal or hemin‐overloaded mice. The iron‐overloaded mouse model was used because it more closely reproduces the conditions found in humans during a *V. vulnificus* infection (Starks et al., 2000; Wright et al., 1981). No difference in 50% lethal dose (LD_50_) was observed between the hemin receptor mutants and the wild‐type strain in normal, non‐iron‐overloaded mice (Table 4). The LD_50_ increased 10,000‐fold for both the *ΔhupA* and *ΔhupAΔhvtA* strains in hemin‐overloaded mice relative to the wild‐type strain. However, the *hvtA* mutant strain exhibited only a marginal attenuation (Table 4). These data indicate that the HupA receptor in the biotype 1 CMCP6 strain is the dominant receptor needed for a fully virulent phenotype in a hemin‐overloaded mouse model (physiological temperature being 37°C). The biotype 2 strain ATCC 33149 (isolated from diseased Japanese eels and most abundantly found in coastal seawater at lower temperatures) was also tested in both normal mice and hemin‐overloaded mice and was found to be avirulent at its highest concentration of 10^8^ cells/ml (data not shown). This is in stark contract to the data presented in Table 4 using the CMCP6 biotype 1 strain. These data suggest that the growth temperature of the bacteria in mice was more important for inducing the *hupA* gene than the *hvtA* gene in the human pathogenic strain (Table 4).

**Table 4:** Virulence of *V. vulnificus* strains in various murine models

*V. vulnificus* strain	LD_50_ a	
	Normal iron level	Hemin‐overloaded model
Wild‐type CMCP6	2 × 10^5^	4
Δ*hvtA*	2 × 10^5^	30
Δ*hupA*	3 × 10^5^	3 × 10^4^
Δ*hvtA*Δ*hupA*	3 × 10^5^	4 × 10^4^

a At least five animals per dilution were inoculated with the indicated strains, and mortality was recorded at 48 hr. LD_50_ value for each strain was calculated as described in Materials and Methods. The data presented here are an average of three biological replicates.

**Figure 5:**
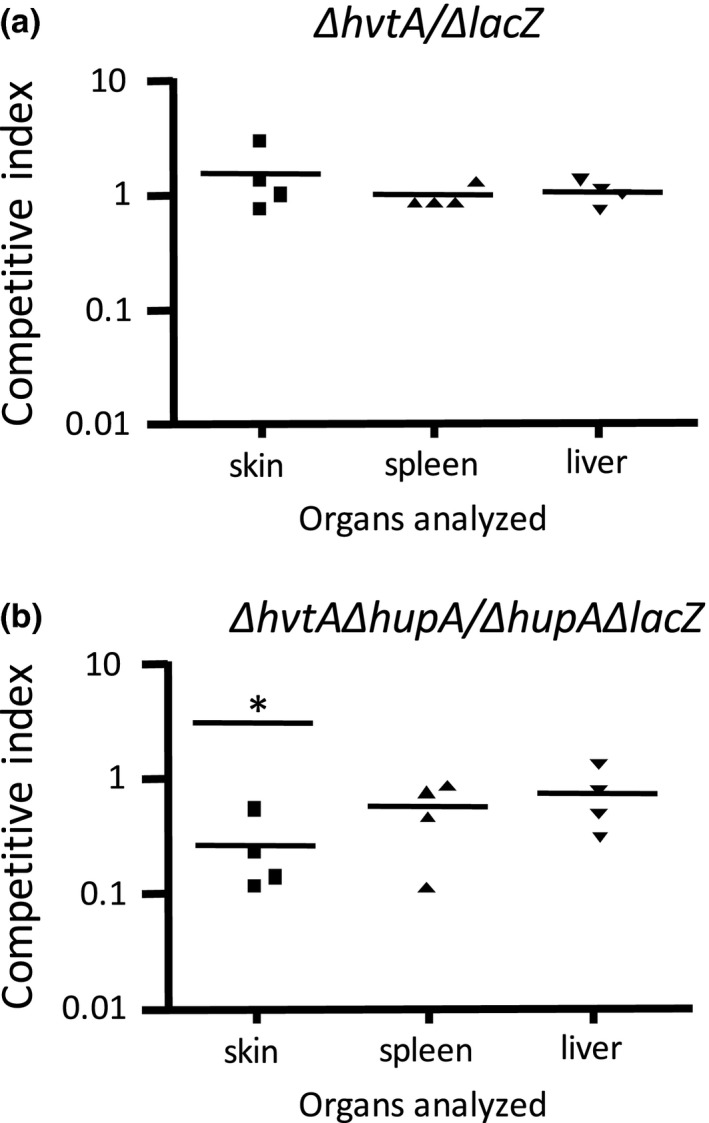
Competitive Index (CI) for hemin transport mutant strains. Mixtures of the strains analyzed (0.1 ml) were injected i.p. into hemin‐overloaded animals. When animals showed signs of disease, organs (skin, spleen, and liver) were removed, homogenized, and bacterial cells were plated on TSAS‐X‐Gal plates. Bars represent the geometric mean CI value for each organ. (a) Δ*hvtA*/Δ*lacZ* (*n* = 4); (b) Δ*hvtA*Δ*hupA*/Δ*hupA*Δ*lacZ* (*n* = 4). Significance (*p < 0.05) was determined through a Student *t* test

In addition to lethality studies, Competitive Index were also generated to extract subtle differences between the virulence of the wild‐type (ALE‐LAC) and *hvtA* mutant (VSSD58) strains. The ability of the two strains to compete for growth in the iron‐overloaded CD‐1 mouse was determined by counting the number of viable bacteria isolated from the skin, spleen, and liver of the host. The CI of *ΔhvtA* to wild‐type did not change dramatically from 1.0, indicating that the *hvtA* mutant strain did not have a growth defect when compared to the wild‐type strain. This was likely because the activity from the product of the *hupA* gene had masked any observable phenotype at this temperature (Figure 5a). To eliminate the effect of the contribution of the *hupA* gene in iron uptake, another CI analysis was performed to compare the *ΔhupAΔlacZ* with the *ΔhvtAΔhupA* strain (Figure 5b). In this comparison, only marginal differences were seen in the spleen and liver. These were not significantly different than a CI score of 1. These results indicate that the *ΔhvtAΔhupA* strain did not have a growth defect compared with the *ΔhupAΔlacZ* strain in these two organs. A small yet significant difference was seen in the skin sample when the *ΔhvtAΔhupA* strain was compared against the *ΔhupAΔlacZ* strain and did significantly vary from a CI score of 1 (Figure 5b). These data indicate that a significant growth defect occurred in the *ΔhvtAΔhupA* strain compared with the *ΔhupAΔlacZ* strain in the skin sample. This indicates a role for *hvtA* in *V. vulnificus* infection when the bacterium is found at the skin of the murine model.

## 4 DISCUSSION

*Vibrio vulnificus* is known to cause severe sepsis in patients who carry abnormally high concentrations of iron in their bloodstream. This organism has developed several iron‐acquisition systems allowing both siderophores and hemin uptake. These systems support its evolution as a facultative human and fish pathogen where infections often result in fatality (Amaro & Biosca, 1996; Gulig et al., 2005; Hor, Chang, Chang, Lei, & Ou, 2000; Tison et al., 1982). Commonly, iron complexes are internalized by bacteria by first being bound by outer membrane receptor proteins. The energy supplied for internalization is produced in the inner membrane through the action of proton motive force (PMF). This energy is then harnessed by the TonB systems and transferred to the respective outer membrane receptor to facilitate the internalization of the ferric complexes (Braun, 1995; Crosa et al., 2004; Postle & Larsen, 2007). In this report, we studied and characterized additional outer membrane proteins in *V. vulnificus* that are necessary for optimal uptake of hemin. This bacterium possesses three distinct TonB systems (TonB1, TonB2, and TonB3) where the TonB3 system was observed to be induced only when bacteria were cultured in human serum (Alice et al., 2008). Here, we demonstrate that HupA and HvtA receptors are dependent on either TonB1 or TonB2, consistent with other iron transport proteins in this organism. The existence of multiple TonB‐dependent hemin‐acquisition systems in human pathogens is not uncommon and has been identified in another closely related Gram‐negative bacterium *V. cholerae* (Mey & Payne, 2001). The apparent redundancy in roles appears to emphasize their importance in establishing virulence inside the host where chances of encountering hemin as a source of iron are high (Helms et al., 1984).

Our analysis of the HupA and HvtA proteins identified and sequenced in the eel pathogen *V. vulnificus* biotype 2 strain (ATCC33149) demonstrates significant homology to the respective proteins in the biotype 1 CMCP6 strain. The HupA protein contains the conserved FRAP motif that has been implicated strongly with receptor function in other iron‐sequestering proteins, particularly in hemin uptake systems. In addition, it includes the partial NPNL sequence downstream of the FRAP motif, including a conserved histidine residue between the two motifs that were shown to participate in hemin binding (Bracken et al., 1999). We found that the HvtA protein in the biotype 2 strain contains motifs that are 75% and 50% identical to the FRAP and NPNL boxes, respectively. This observation was consistent with the notion that the NPNL motif is not well conserved in hemin receptors from other *Vibrio* species (Mey & Payne, 2001). While the *hupA* gene in the CMCP6 strain was shown to be monocistronic (Litwin & Byrne, 1998), we found that the *hvtA* gene is encoded in a polycistronic message present on the second chromosome. RT‐PCR analysis demonstrated that the putative start site of the operon was upstream of the *ptrB* gene that precedes the *hvtA* gene. The results also indicated that VV21546 was not part of the operon, whereas VV21550‐2 was co‐transcribed with the *hvtA* gene. This operon exhibits significant homology to the *hutR* containing operon in *V. cholerae*, with the functions of the products of genes VV21550‐2 remaining unknown.

In this study, we confirmed experimentally that the expression of the *hvtA* operon in the CMCP6 biotype 1 strain was dependent on iron concentration with Fur regulation as hypothesized by Gulig et al. (2010). A potential binding site for the Fur protein (5′‐gtaaatgataattgatgt‐3′) was identified in the putative promoter region of the *hvtA* operon that shares 63% identity with the consensus Fur binding sequence (5′‐gataatgataatcattatc‐3′; Lavrrar & McIntosh, 2003). The *fur* mutant strain could not modulate expression levels of examined genes in iron‐rich versus iron‐limiting conditions. This is consistent with the regulatory patterns of iron‐acquisition genes in different Gram‐negative bacteria including *V. vulnificus* which are regulated by Fur in response to this element (Escolar et al., 1999; Pajuelo et al., 2014).

In addition to being regulated in response to iron, transcriptional analysis determined that both the *hupA* and *hvtA* genes exhibit temperature‐dependent expression in biotype 1 and biotype 2 strains. This type of temperature‐dependent transcriptional regulation was previously reported for the *virF* gene in Yersiniae, whose product is a transcriptional regulator of the AraC family and plays a vital role in controlling the expression of virulence factors (Falconi, Colonna, Prosseda, Micheli, & Gualerzi, 1998; Konkel & Tilly, 2000; Wattiau & Cornelis, 1994). In addition, recent reports have identified RNA‐mediated thermoregulation of iron‐acquisition genes in *Shigella* (Kouse, Righetti, Kortmann, Narberhaus, & Murphy, 2013; Wei, Kouse, & Murphy, 2017). This is particularly significant because *V. vulnificus* infecting humans (biotype 1) or eels (biotype 2) must regulate gene expression in dramatically different temperatures depending on the host organism. The ambient temperature in the host environment likely controls the upregulation of certain virulence genes essential for pathogenesis. Elevated mRNA levels of the *hupA* and *hvtA* genes in both biotype 1 at 37°C and biotype 2 at 25°C indicate the significance of each gene at a particular temperature. This fact suggests a possible adaptation of the hemin receptors in response to the range of temperatures exhibited by susceptible hosts. We hypothesize the presence of a possible transcriptional switch that opportunistically interacts with the active promoters depending on DNA conformation influenced by environmental temperature.

Our studies also demonstrated that a mutation in the *hupA* gene resulted in a significant reduction in virulence compared with the wild‐type CMCP6 biotype 1 strain in hemin‐overloaded mice. Deletion of the *hvtA* gene resulted in a strain that was only marginally attenuated. This observation is consistent with the virulence experiments explained in Pajuelo et al. where similar results were obtained with the *V. vulnificus* biotype 2 strain (CECT4999) when tested in eels and mice (Pajuelo et al., 2014). It is interesting to note that the CECT4999 strain used in the study is a Spanish isolate that is pathogenic for both mice and eels. The ATCC33149 biotype 2 strain however has not been found to exhibit any sign of mice pathogenicity in our experiments or in earlier studies (Biosca, Oliver, & Amaro, 1996). It would be interesting to study the virulence of the hemin receptor mutants of this strain in eels in order to determine their importance. Attempts to generate individual hemin receptor mutants in the ATCC33149 biotype 2 strain have been unsuccessful thus far.

*Vibrio vulnificus* causes severe septicemia and it is reasonable to postulate that hemin acquisition serves a major role in the pathogenesis of the human host, where the majority of available iron in serum is sequestered by hemin and hemoglobin in red blood cells. However, it appears that the organism triggers the expression of specific hemin receptors at different temperatures. The significance of the *hupA* gene as a virulence factor in the murine model at the physiological temperature of 37°C is consistent with the observation that its expression was highly induced at elevated temperatures in iron‐limiting conditions. Alternately, it is possible that the *hvtA* gene has evolved to play a more important role in certain *V. vulnificus* strains whose primary host is eels, where the growth temperature is closer to 25°C in coastal seawaters. Indeed, *hvtA* expression was highly upregulated in the biotype 2 strain at 25°C, and was not induced to the extent of *hupA* at 37°C in either biotype. The fact that the biotype 2 ATCC33149 strain was avirulent in the murine model suggests that temperature plays a vital role in the expression of virulence factors in the bacterium. In addition, the *hvtA* gene may be of real importance when an open wound infection is exposed to *V. vulnificus* contaminated waters. With the lower temperature found at the wound site in open waters, *hvtA* expression may be important in early‐stage infections acquiring hemin from the host.

Additionally, in our CI studies, we only saw significant growth differences between our *ΔhvtAΔhupA* strain compared to our *ΔhupAΔlacZ* strain in the skin sample. The *ΔhvtAΔhupA* strain did not grow as well as the *ΔhupAΔlacZ* strain in this sample. Skin is generally found to be at a lower temperature (34°C in humans, 33.6°C in mice) compared with the spleen and liver (37° in humans, 36.6° in mice; Mei et al., 2018; Reitman, 2018). Again, this lower temperature found at a potential wound site could help in early‐stage infections in acquiring hemin from a host, suggesting a role for *hvtA* in infections at lower temperatures.

The differential temperature response of these hemin receptor‐encoding genes confirms the role of a yet unknown regulatory switch that controls the expression of these genes in response to iron and temperature. Interestingly, Pajuelo et al. reported that a biotype 2 serovar CECT4999 strain showed equal levels of virulence when tested in mice and eels where hemin receptor *hupA* and vulnibactin receptor *vuuA* served as the major virulence factors (Pajuelo et al., 2014). The *hvtA* (named *hutR* in strain CECT4999) single mutant behaved similarly to the wild‐type in either hosts suggesting that *hupA* and *vuuA* are host‐nonspecific virulence genes, but *hvtA* is not (Pajuelo et al., 2014).

Bacterial pathogenesis is a complex energy‐intensive process involving multiple factors that not only contribute to disease progression but also promote successful replication inside the host organism. Pathogens have evolved to distinguish between environments and ensure that a large number of virulence genes are expressed only when the bacterium is invading host tissues. Changes in temperature, nutrients, osmolarity, iron, and other ion concentrations serve as cues that promote preferential expression of virulence genes necessary for survival. Our studies indicate that a dual regulation of the hemin receptors exists that responds to both iron concentration and temperature and that this regulation is critical to the pathogenesis of *V. vulnificus*. Where one of the receptors encoded by the *hupA* gene is heavily expressed at an elevated temperature, the other, produced from the *hvtA* gene, is likely more biologically relevant at a lower temperature as observed from its expression profile. These data suggest that the receptors have unique regulatory features associated with them. Future studies will incorporate the identification and characterization of the regulatory switch in order to unravel the mechanism of gene regulation in this bacterium. Understanding the molecular basis for this dual regulation will provide a platform for understanding virulence gene regulation in other pathogens that alternate between hosts with variable ambient temperatures.

## CONFLICT OF INTERESTS

The authors have no conflicts of interest to disclose.

## AUTHOR CONTRIBUTIONS

S.D and R.J.K conceptualized the data, curated the data, involved in formal analysis, investigated the data, contributed to methodology, administered the project, involved in resource management, provided software, supervised the data, validated the data, visualized the manuscript, wrote the original draft, and reviewed and edited the manuscript. R.J.K acquired the funding.

## ETHICS STATEMENT

All manipulations of mice were approved by the Institutional Animal Care & Use Committee (IACUC) at the Oregon Health Science University, protocol #A802.

## Data Availability

All data are provided in full in the results’ section of this paper apart from the full protein sequences used to create alignments. These sequences are available at the National Center for Biotechnology Information (NCBI) at http://www.ncbi.nlm.nih.gov/genbank/ under accession numbers WP_011082397 (*V. vulnificus* CMCP6, HvtA), ASJ40441 (*V. vulnificus* ATCC33149, HvtA), WP_032480606 (*V. cholerae*, HutR), AAO07241 (*V. vulnificus* CMCP6, HupA), ASJ40757 (*V. vulnificus* ATCC33149, HupA), and WP_080007439 (*V. cholerae*, HutA).
